# Comparison of vestibular aqueduct visualization on computed tomography and magnetic resonance imaging in patients with Ménière’s disease

**DOI:** 10.1186/s12880-024-01275-8

**Published:** 2024-04-22

**Authors:** Kaijun Xia, Ping Lei, Yingzhao Liu, Cen Chen, Hui Pan, Yangming Leng, Bo Liu

**Affiliations:** 1 Department of Otorhinolaryngology-Head and Neck Surgery, Union Hospital, Tongji Medical College, Huazhong University of Science and Technology, Wuhan, 430022 China; 2 Department of Radiology, Union Hospital, Tongji Medical College, Huazhong University of Science and Technology, Wuhan, 430022 China

**Keywords:** Ménière’s disease, Vestibular aqueduct, Magnetic resonance imaging, Computed tomography

## Abstract

**Background:**

The vestibular aqueduct (VA) serves an essential role in homeostasis of the inner ear and pathogenesis of Ménière’s disease (MD). The bony VA can be clearly depicted by high-resolution computed tomography (HRCT), whereas the optimal sequences and parameters for magnetic resonance imaging (MRI) are not yet established. We investigated VA characteristics and potential factors influencing MRI-VA visibility in unilateral MD patients.

**Methods:**

One hundred patients with unilateral MD underwent MRI with three-dimensional sampling perfection with application optimized contrasts using different flip angle evolutions (3D-SPACE) sequence and HRCT evaluation. The imaging variables included MRI-VA and CT-VA visibility, CT-VA morphology and CT-peri-VA pneumatization.

**Results:**

The most frequent type of MRI-VA and CT-VA visualization was invisible VA and continuous VA, respectively. The MRI-VA visibility was significantly lower than CT-VA visibility. MRI-VA visibility had a weak positive correlation with ipsilateral CT-VA visualization. For the affected side, the MRI-VA visualization was negatively correlated with the incidence of obliterated-shaped CT-VA and positively with that of tubular-shaped CT-VA. MRI-VA visualization was not affected by CT-peri-VA pneumatization.

**Conclusion:**

In patients with MD, the VA visualization on 3D-SPACE MRI is poorer than that observed on CT and may be affected by its osseous configuration. These findings may provide a basis for further characterization of VA demonstrated by MRI and its clinical significance.

**Supplementary Information:**

The online version contains supplementary material available at 10.1186/s12880-024-01275-8.

## Background

Ménière’s disease (MD) is a common peripheral vestibular disorder histopathologically characterized by idiopathic endolymphatic hydrops (ELH), with episodic vertigo, fluctuating hearing loss, tinnitus, or aural fullness as its main clinical symptoms. Although its etiology and pathophysiological mechanisms have not yet been fully elucidated, it is currently believed that MD is associated with disturbed homeostasis of the inner ear, for instance, excessive production and/or impaired absorption of endolymphatic fluid [1]. Many factors may be involved in this pathological process, among which, anatomical abnormalities have been suggested as a predisposing factor underlying impaired endolymphatic drainage [1–3]. Besides ELH, histological studies have revealed atrophy of the endolymphatic sac (ES), hypoplasia of the vestibular aqueduct (VA), and narrowing of the lumen of the endolymphatic duct (ED) in MD patients [4–6]. Until now, many radiological literatures also have highlighted these pathological findings associated with ES, ED, or VA in patients with MD [2, 7–10].

The VA is a bony canal running from the common crus and opening on the posterior surface of the petrous portion of the temporal bone. The ED takes its origin from the utricle and the saccule in the vestibule and then passes posteriorly into the skeletal tunnel of the VA with transformation to ES. The ES is simply a specialized portion of the posterior fossa dura that contains the resorptive epithelium of the membranous labyrinth [11]. Atrophy of the ES, hypoplasia of the VA and narrowing of the lumen of the ED has been demonstrated in MD patients by histological investigations [4–6]. Radiological studies have described a correlation between the MD and the lack of a visible ED or VA by using computed tomography (CT), 3D-cone beam CT and magnetic resonance imaging (MRI) [2, 10, 12, 13]. The first-choice imaging technique to visualize the VA is high resolution CT (HRCT), which typically shows axially a small osseous notch at the posterior margin of the petrous bone, mostly at the level of the horizontal semicircular canal (SCC) or its adjacent superior and inferior levels [14, 15]. Many MRI sequences have been used to evaluate the ED or VA, including the three-dimensional fluid attenuated inversion recovery (3D-FLAIR), 3D sampling perfection with application optimized contrasts using different flip angle evolutions (3D-SPACE), etc [3, 16]. Lorenzi et al*.* reported that the ED appeared to be statistically less visible on T2-weighted fast spin-echo sequence with fat suppression in patients with MD [17]. Patel et al*.* reported that lack of VA visualization on T2-weighted gradient echo sequence without contrast was statistically significant in MD patients compared to the general population [18]. Attyé et al*.* detected VA abnormalities in both ears of patients with unilateral MD by using 3D-FLAIR sequence [16]. Recently, using the 3D-SPACE sequence, we also found anatomical differences in endolymphatic drainage system between MD and vestibular migraine in terms of VA visibility and distance between the vertical part of the posterior SCC and the posterior fossa [19]. Until now, the optimal sequences and parameters remain an open question. Moreover, it is also warranted to investigate the factors influencing MRI-VA visualization, such as osseous VA morphology.

This retrospective study was designed to compare radiological VA visibility using CT and MRI simultaneously in unilateral MD patients. The purpose of this study was to further explore the effect of osseous anatomical variables on visibility of MRI-VA in patients with MD, and the clinical value of VA radiological evaluation in MD was also discussed.

## Methods

### Study population

This retrospective study included a total of 100 patients with definite unilateral MD who attended the outpatient department of Otorhinolaryngology, Union Hospital, Tongji Medical College, Huazhong University of Science and Technology between September 2012 and December 2022. The diagnosis of MD was established following the diagnostic criteria proposed by the American Academy of Otolaryngology-Head and Neck Surgery (AAO-HNS) in 1995 [20]. For all patients, a detailed history inquiry, otoscopy, audio-vestibular evaluations, and imaging examination were conducted for differential diagnosis. MD stage was determined based on the hearing level of the affected side, according to the AAO-HNS guidelines (1995) [20].

The exclusion criteria were: (1) bilateral MD; (2) middle or inner ear anomaly; (3) middle or inner ear infections (otitis media, mastoiditis, labyrinthitis etc.); (4) retro-cochlear lesions (vestibular schwannoma, internal acoustic canal stenosis etc.); (5) having received previous ear surgery or intratympanic injections; (6) head trauma; (7) systemic diseases; (8) disorders of central nervous system (vestibular migraine, multiple sclerosis, cerebellar infarction, etc); (9) the interval between CT and MRI examination over 1 month.

This study was conducted according to the tenets of the Declaration of Helsinki. Each patient signed informed consent. Ethics approval was obtained from the ethical committee of Union Hospital, Tongji Medical College, Huazhong University of Science and Technology.

### Radiological evaluations

The imaging protocols of CT have been described in detail in our previous article [21]. Briefly, all patients underwent a spiral CT scan (Somatom Defnition AS+, Siemens, Germany) while supine in a craniocaudal direction. The scan plane was parallel to the orbitomeatal line. The following parameters were used: tube voltage, 120 kV; CASE Dose 4D quality reference mAs, 180 mAs; slice thickness, 0.6 mm; slice collimation, 128×0.6 mm; pitch, 0.5; field of view, 150mm; reconstruction increment, 0.3 mm; reconstruction kernel, H60s. The MRI examination was performed using the Verio or Magnetom Trio 3T scanners (Siemens, Erlangen, Germany) with a 12-element phased array coil. T1-weighted and T2-weighted spin-echo images were acquired. The visualization of VA was evaluated using 3D-SPACE with the following parameters: repetition time (TR), 1000 msec; echo time (TE), 135 msec; field of view (FOV), 200 × 200 mm^2^; slice thickness, 0.5 mm; matrix, 384 × 384; averages, 2; bandwidth, 289 Hz/Px.

All MRI and CT images were transferred to and analyzed on a picture archiving and communication system (PACS) workstation (Carestream Client, Carestream Health, Rochester, NY, USA). Two senior neuroradiologist assessed the radiological data independently, who were blinded to the clinical information. In this study, VA visualization was evaluated mainly on MRI and CT axial images from the vestibule to the posterior edge of the temporal bone (MRI-VA visibility and CT-VA visibility, respectively), as well as on sagittal and coronal images. Other radiological indices based on CT examination included the VA morphology and peri-VA pneumatization.

**Fig. 1:**
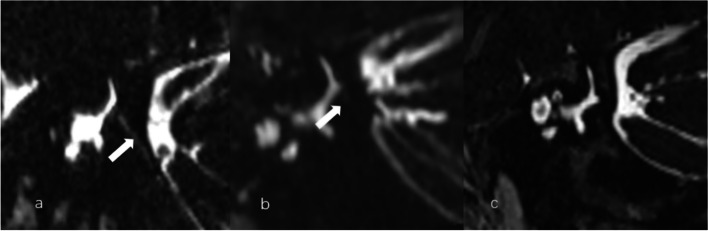
Vestibular aqueduct (VA) grading on the Pöschl planes on three-dimensional sampling perfection with application optimized contrasts using different flip angle evolutions (3D-SPACE). **a** Grade A with a continuous VA (arrow). **b** Grade B with a discontinuous VA (arrow). **c** Grade C with a no visible VA

The visibility of VA in MRI was graded as [16]: Grade A, the presence of VA was confirmed with a linear duct starting from the posterior edge of the temporal bone to the vestibule; Grade B, a discontinuous VA; and Grade C, a complete absence of visible VA. The typical images were acquired in the Pöschl plane (oblique 45° plane) (Fig. 1), for the Pöschl plane is parallel with the longitudinal axis of VA, thus not affected by the axial obliquity of the scan acquisition or obliquity of VA itself.

**Fig. 2:**
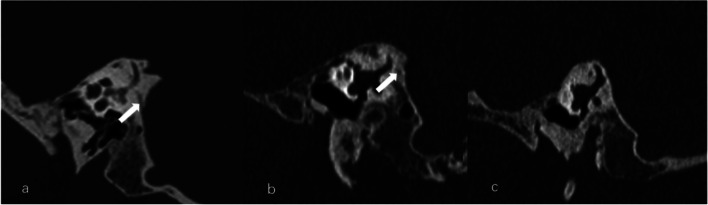
The visibility of vestibular aqueduct (VA) grading on the Pöschl planes on temporal bone CT. **a** Grade 0 with a continuous VA (arrow). **b** Grade I with a discontinuous VA (arrow). **c** Grade II with a complete ossification of VA

**Fig. 3:**
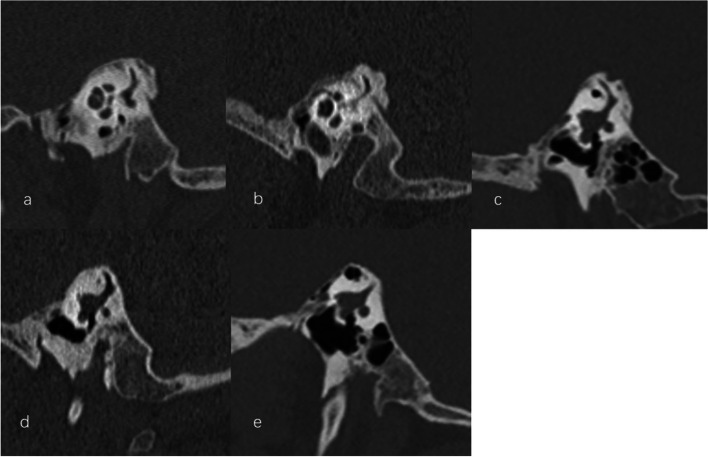
Various types of vestibular aqueduct (VA). **a** funnel type. **b** tubular type. **c** filiform type. **d** hollow type. **e** obliterated type

**Fig. 4:**
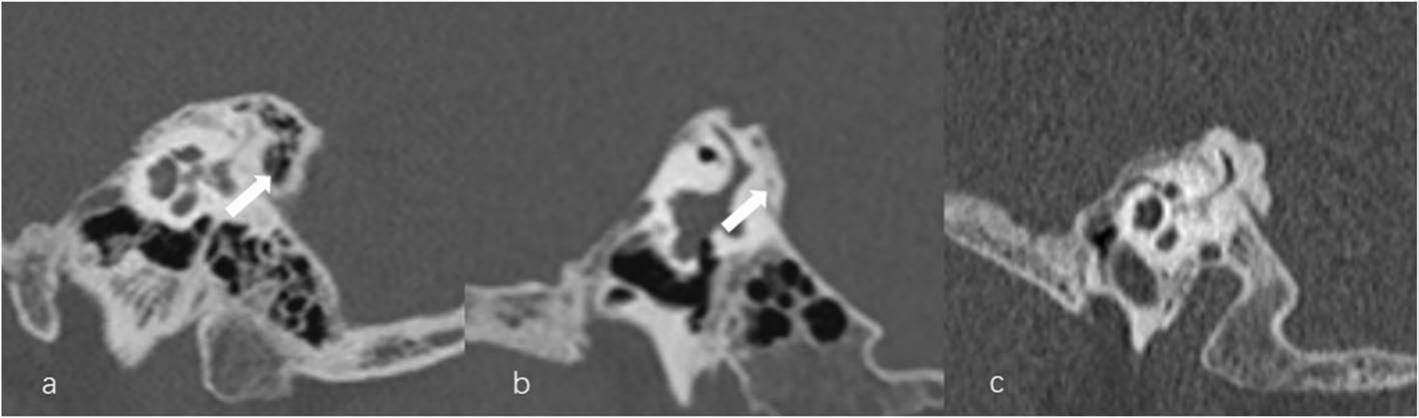
Different types of peri-aqueductal pneumatization. **a** Large-cell pneumatization (arrow) in the vicinity of the aqueduct. **b** Small-cell pneumatization (arrow) in the vicinity of the aqueduct. **c** Absence of air cell

CT-VA visibility of was graded as [2]: Grade 0, continuous VA; Grade I, discontinuous VA; Grade II, a complete absence of visible VA (Fig. 2). According to Yamane et al’s criteria, the morphology of VA in the 45°oblique(Pöschl) plane were classified as funnel, tubular, filiform, hollow, and obliterated type [12] (Fig. 3). Peri-VA pneumatization were classified into: large-cell pneumatization (Grade 1), small-cell pneumatization (Grade 2), and absence of air cells (Grade 3) in the vicinity of the VA, as described by Stahle and Wilbrand (Fig. 4) [22].

### Statistical analysis

The data were analyzed and processed by SPSS 25.0 statistical software. Continuous variables were expressed as mean ± standard deviation or median (quartile). Categorical variables and grade variables were expressed as frequency (percentage). Shapiro-Wilk test was conducted to assess the normality of data. We performed Wilcoxon sign-rank test and McNemar-Bowker test to compare the grade variables and the categorical variables, respectively, of two related samples. The correlation between grade variables and binary variables was analyzed by Mantel-Haenszel Chi square test. The correlation between grade variables and continuous variables was analyzed by Spearman correlation analysis. Kappa value was used to describe the degree of concordance between radiologists for count data and grade data, respectively. The interpretation of concordance was as follows: kappa ≤ 0.20, poor; 0.21<kappa ≤ 0.40, fair; 0.41<kappa ≤ 0.60, moderate; 0.61<kappa ≤ 0.80, good; 0.81<kappa ≤ 1.0, excellent. *P* < 0.05 was considered as statistically significant.

## Results

A total of 100 patients with unilateral MD were included in this study, of whom 37 were male and 63 were female. The median age was 51.5 years (44.25, 57) and the median duration of disease was 1.5 (0.5, 3) years. Unilateral MD affected left ears in 58 cases and right ears in 42 cases. The Ménière ‘s stage was graded as Stage I in 11 (11.0%) cases, Stage II in 20 (20.0%) cases, Stage III in 55 (55.0%) cases, and Stage IV in 14 (14.0%) cases. Correlational analysis revealed no significant association between the Ménière ‘s stage and MRI-VA visibility (${r}_{s}$=0.093, *p* =0.358), CT-VA visibility (${r}_{s}$=0.086,* p* =0.393), peri-VA pneumatization (${r}_{s}$=-0.003, *p* =0.979), and VA morphology (${\upchi }^{2}$=16.488, *p* =0.170).

### Radiological assessment of VA in patients with unilateral MD

Good to excellent inter-observer agreement was found in our radiological evaluations regarding to MRI-VA visibility (kappa=0.823), CT-VA visibility (kappa=0.814), peri-VA pneumatization (kappa=0.920), and VA morphology (kappa=0.836). Additional file 1 shows detailed clinico-radiological data of patients with unilateral MD assessed independently by L.P and C.C.

As for MRI-VA visibility, 11 (11.0%) unilateral MD patients presented with continuous VA (Grade A), 20 (20.0%) cases with discontinuous VA (Grade B), and 69 (69.0%) cases with absent VA (Grade C) in the affected side. In the non-affected side, 18 (18.0%) cases were graded as Grade A, 25 (25.0%) cases as Grade B, and 57 (57.0%) cases as Grade C. The distribution of MRI-VA visualization was statistically different between the affected and non-affected side (Z = -2.234, *p* = 0.025) (Table 1). CT measurement identified continuous VA (Grade 0) in the affected side of 72 (72.0%) unilateral MD patients and discontinuous VA (Grade 1) in 28 (28.0%) cases. In the non-affected side, the level of CT-VA visibility was rated as Grade 0 in 75 cases (75.0%) and Grade 1 in 25 case (25.0%). There was no statistical difference in the distribution of CT-VA visualization between the affected and non-affected side (Z = -0.655, *p* = 0.513) (Table 1). The MRI-VA visibility was considerably poorer than CT-VA visibility in both the affected (Z = -8.314, *p* < 0.001) and non-affected side (Z = -7.814, *p* < 0.001) respectively.

**Table 1:** Interaural comparison of radiological variables in unilateral MD patients

Radiological variables		Affected ears (*n*=100)	Non-affected ears (*n*=100)	z/${x}^{2}$	*p*
MRI-VA visibility	Grade A	11(11.0%)	18(18.0%)	z=-2.234	p=0.025
	Grade B	20(20.0%)	25(25.0%)		
	Grade C	69(69.0%)	57(57.0%)		
CT-VA visibility	Grade 0	72(72%)	75(75.0%)	z=-0.655	p=0.513
	Grade I	28(28.0%)	25(25.0%)		
	Grade II	0(0.0%)	0(0.0%)		
Type of CT-VA	funnel type	17(17.0%)	17(17.0%)	${x}^{2}$=6.604	p=0.471
	tubular type	28(28.0%)	31(31.0%)		
	filiform type	32(32.0%)	38(38.0%)		
	hollow type	1(1.0%)	1(1.0%)		
	obliterated type	22(22.0%)	13(13.0%)		
Peri-VA pneumatization	Grade 1	20(20.0%)	22(22.0%)	z=-0.499	p=0.618
	Grade 2	21(21.0%)	20(20.0%)		
	Grade 3	59(59.0%)	58(58.0%)		

*n* number of ears, *MD* Meniere’s Disease, *VA* vestibular aqueduct

CT examination showed that, in the affected side, VA shape was classified as funnel type in 17 unilateral MD cases (17.0%), tubular type in 28 cases (28.0%), filiform type in 32 cases (32.0%), hollow type in 1 case (1.0%), and obliterated type in 22 cases (22.0%). In the non-affected side, 17 cases (17.0%) exhibited funnel type VA, 31 cases (31.0%) tubular type, 38 cases (38.0%) filiform type, 1 case (1.0%) hollow type, and 13 cases (13.0%) obliterated type. No significant difference in the distribution of VA shape was found between the affected side and the non-affected side (${x}^{2}$= 6.604, *p* = 0.471) (Table 1).

For the degree of peri-VA pneumatization, large-cell pneumatization (Grade 1) was observed in 20 cases (20.0%), small-cell pneumatization (Grade 2) in 21 cases (21.0%), and no pneumatization (Grade 3) in 59 cases (59.0%) in the affected side. In the non-affected side, 22 cases (22.0%) were graded as Grade 1, 20 cases (20.0%) as Grade 2, and 58 cases (58.0%) as Grade 3. There was no significant difference in the grading of peri-VA pneumatization between the affected and non-affected side (Z = -0.499, *p* = 0.618) (Table 1).

### Correlation between osseous anatomical variables and visibility of MRI-VA

Correlation analysis showed a weak positive correlation between MRI-VA visualization and ipsilateral CT-VA visualization in patients with unilateral MD in both the affected side (${r}_{s}$ = 0.285, *p* = 0.004) and the non-affected side (${r}_{s}$ = 0.243, *p* = 0.015), respectively.

Significant correlations were observed between CT-VA morphology and ipsilateral VA visualization based on both CT and MRI assessment. In the MD-affected ears, MRI-VA visibility was negatively correlated with the incidence of obliterated-shaped CT-VA (${x}^{2}$= 6.523, *p* = 0.011) and positively correlated with that of tubular-shaped CT-VA (${x}^{2}$= 5.552, *p* = 0.018). Similarly, poor CT-VA visibility was associated with the incidence of obliterated-shaped CT-VA (${x}^{2}$= 6.771, *p* = 0.009). In the non-affected ears, MRI-VA visibility was positively related to the occurrence of tubular-shaped CT-VA (${x}^{2}$= 6.394, *p* = 0.011) and CT-VA visualization was negatively related to that of obliterated-shaped CT-VA (${x}^{2}$= 10.640, *p* = 0.001).

MRI-VA visibility was not affected by ipsilateral peri-VA pneumatization in patients with unilateral MD in either the affected side (${r}_{s}$ = 0.109, *p* = 0.278) or the non-affected side (${r}_{s}$ = -0.004, *p* = 0.970). No correlation was found between CT-VA visibility and ipsilateral peri-VA pneumatization in the affected side (${r}_{s}$ = 0.028, *p* = 0.785), whereas a weak positive correlation was noted between these two variables in the non-affected side (${r}_{s}$ = 0.314, *p* = 0.001).

## Discussion

### Visibility of VA in HRCT and in MRI

Osseous VA could be clearly depicted on CT images, whereas the VA itself cannot be observed directly on MRI but only inferred indirectly through the high signal of its contents, i.e., the vessels or fluid within the VA. This study showed that CT-VA visibility was superior to MRI-VA visibility in the same cohort of patients with unilateral MD. Recent literatures have reported variable results regarding to the VA visibility using CT or MRI. The proportion of VA non-visualization on CT was 30% by Grosser et al*.* [13] and 42.8% by Mainnemarre et al*.* [2], respectively. The proportion of VA non-visualization on MRI ranges from 20.6% to 76.67% [16, 17, 23, 24] .

VA is an essential component of the endolymphatic outflow system, and abnormalities in its morphology or function play an important role in the pathogenesis of MD. Recently, several histopathological and radiological studies have focused on this outflow system of the inner ear in patients with MD. In a histopathological study of human temporal bones, Michaels et al*.* described a thin, highly vascular layer of mineralized cartilage within VA, surrounding most of the course of ED [25]. By studying archival temporal bone sections and a surgical specimen, Linthicum et al*.* discovered the presence of a small network of channels (called “peri-endolymphatic channel system”) surrounding the human VA that extend from the proximal cisternal area of the ES to the supporting tissue of the saccule and utricle [26]. The authors speculated that this channel system may be involved in endolymph resorption. More recently, Nordström et al. performed a micro-CT and synchrotron phase contrast imaging (SR-PCI) study with 3D reconstructions to demonstrate the organization of the peri-endolymphatic channel system and its relation to the surrounding draining veins [27]. SR-PCI and 3D reconstructions of the temporal bones revealed a rich plexus of channels which surrounds the human VA containing ED. This vascular plexus is composed of sinusoidal tissue channels, lymphatics, and sinusoidal veins. Due to the relatively low spatial resolution of MRI and the fibrotic changes within the richly vascularized peri-saccular loose connective tissue [2, 28], the MRI-VA visibility demonstrated by 3D-SPACE sequences was poorer than CT-VA visibility in patients with MD.

Using MRI with both non-enhanced and enhanced 3D-FLAIR sequences in MD patients, Attyé et al*.* suggested that the invisibility of MRI-VA was due to the relatively low spatial resolution and disturbed endolymphatic composition caused by elevated calcium ion level, while the continuous visibility of VA probably corresponded to the highly vascular layer within the otic capsule surrounding ED [16]. MRI evaluation of the VA warrants further exploration, and the optimal imaging protocols and parameters as well as the MRI signal in the VA region are awaited to establish.

In this study, there was a slight difference in MRI-VA visualization distribution between the affected and non-affected side in unilateral MD patients, and VA was smaller and more difficult to visualize (higher incidence of Grade C) in the affected ear. On the other hand, no interaural difference was observed in the distribution of CT-VA visualization. The MRI findings in this series were inconsistent with our previous findings, which demonstrated no interaural difference of MRI-VA visibility in patients with unilateral MD using the same MRI sequence [3, 19]. Using 3D-FLAIR MRI, the narrowing of the VA was also observed symmetrically in both ears of unilateral MD patients [16]. However, in an earlier radiological study, Tanioka et al*.* found that patients with unilateral MD had less VA visibility in the affected side than in the unaffected side during the vertigo attack using three-dimensional Fourier transformation fast low angle shot (3DFT-FLASH) MRI sequence [10]. Thus, the discrepancy of the present results with previous studies may be attributed to the different cases included and patients’ status of attack.

### Association between osseous anatomical variables and MRI-VA visibility

To date, the mechanism underlying MRI-VA visualization has not been fully elucidated, and the relevant factors impacting MRI-VA visualization need to be further investigated. We found that, the CT-VA visibility and the CT-VA morphology correlated with the MRI-VA visibility, whereas the degree of peri-VA pneumatization did not, which suggested that some osseous anatomical variable may influence the MRI-VA visibility. CT-VA visibility is one of radiographic variables that has been extensively studied, and numerous studies have found significantly reduced VA visibility in patients with MD [2, 13, 21]. Our findings that CT-VA visibility correlated with MRI-VA visibility may pave the way for further characterization of VA presence on MRI and its clinical significance. Furthermore, most recently, using combination of CT and gadolinium-enhanced MRI (Gd-MRI) of the inner ear, the association between visualization of the VA and ELH *in vivo* can be explored in patients with MD. Nonvisualization of VA has been reported to achieve a positive predictive value of 93.1% for predicting the saccular hydrops [2]. Grosser et al*.* also demonstrated a close association between the invisibility of CT-VA and the degree of cochlear hydrops *in vivo* [13].

In addition, our results suggested that morphological variants of the VA, such as obliterated type of VA, may hinder visualization of VA in the MD-affected ear by MRI assessment, which were consistent with Yamane et al*.*’s and our previous findings. Yamane et al. found that the obliterated type of VA is characteristic in the MD affected ears [12]. Our previous study also exhibited a higher incidence of obliterated phenotypes of CT-VA in MD ears (22.1%) than in control ears (6.6%) [21]. The present findings suggest that MRI-VA visualization is reduced in MD patients, which may be resulted from the low-resolution of obliterated type of VA on MR images.

Although CT-VA visibility is higher than MRI-VA visibility, our preliminary results suggested that the bony configuration of VA may affect MRI-VA morphology in patients with MD. Other factors that may possibly influence MRI-VA visibility in MD patients, such as disease duration, clinical stage, autoimmunity, etc., warrant further investigation in the future.

### Implication of VA imaging evaluation on clinical features of MD

It is widely accepted that ES plays multiple roles in the pathogenesis in ELH [29–32]. Recently, VA morphology has been reported to correlate with ES pathologies, which may contribute to the heterogeneity of clinical features and therapeutic response in MD. The angle between the VA proximal to the vestibule and the distal VA (angular trajectory of the VA, ATVA) has been identified as a potential radiographic marker for ES pathologies (endotypes), i.e*.*, degeneration and hypoplasia, in MD patients [33], which are closely linked to distinct clinical features (phenotypes) of MD, such as frequency of vertigo attack, severity of vestibular dysfunction, progression to bilateral MD, etc [34, 35]. The outcome of endolymphatic duct block surgery in MD patients could also be predicted by extraosseous ES pathology [36]. The above findings suggest that a more sophisticated imaging evaluation allows us to better understand the function of the VA or endolymphatic drainage system and may offer new opportunities for precision medicine strategies in MD patients based on different ES endotype. Most recently, de Pont et al*.* measured the ATVA with 3D FLAIR MRI in patients with MD and found that sub-group of hypoplasia endotype is associated with longer disease duration, higher prevalence of bilateral involvement, and trend toward a male preponderance [23]. Therefore, the correlation between the MRI-VA morphology and clinical features in MD patients warrants further in-depth investigation, which may provide new insight into the clinical significance of MRI-VA morphology.

## Strength and limitation

To the best of our knowledge, this study, for the first time, assessed the association between the MRI-VA visibility and CT-VA morphologies. HRCT is the first-choice imaging modality for evaluation of VA morphology or configuration, which is superior to MRI because of the relatively low resolution and liquid disturbance in VA region. However, the osseous configuration of VA could significantly affect VA visibility on MRI using 3D-SPACE sequences. Further investigations are warranted to establish the optimal sequences and parameters for evaluation of VA by MRI.

This study has several limitations. First, this was a retrospective study and healthy controls were not included. Therefore, the association between MRI-VA visibility and CT-VA morphologies in healthy subjects remains to be investigated. Second, patients with bilateral MD were not included in this study. Previous studies have suggested distinct pathologies of the endolymphatic drainage system between patients with bilateral MD and those with unilateral MD [35]. Using ATVA measurement, Bächinger et al*.* showed a higher frequency of bilateral MD in the ES hypoplasia group (29.4%) than in the ES degeneration group (5.5%) [34]. Third, Gd-enhanced MRI was not performed in this study, which was not included in the standard diagnostic workup in our institute. Simultaneous MRI-VA visualization and Gd-enhanced MRI of the inner ear would help elucidate the relationship between ELH and the anatomic and functional alterations of the endolymphatic drainage system. Therefore, larger prospective controlled studies based on sub-typing diagnosis with long-term follow-up are needed to better understand the mechanisms and clinical relevance of MRI-VA visualization in patients with MD.

## Conclusions

In patients with MD, the VA visualization on 3D-SPACE MRI is poorer than that observed on CT and may be affected by its osseous configuration. These findings may provide a basis for further research into the clinical significance of MRI-VA visualization. Further investigations are warranted to establish the optimal sequences and parameters for evaluation of VA by MRI.

### Supplementary Information

**Supplementary Material 1.**

## Data Availability

All data generated or analyzed during this study are included in this published article and its supplementary information files.

## Abbreviations

3D-FLAIR: three-dimensional fluid attenuated inversion recovery
3DFT-FLASH: three-dimensional Fourier transformation fast low angle shot
3D-SPACE: 3D sampling perfection with application optimized contrasts using different flip angle evolutions
AAO-HNS: American Academy of Otolaryngology-Head and Neck Surgery
ATVA: Angular trajectory of the vestibular aqueduct
CT: Computed tomography
ED: Endolymphatic duct
ELH: Endolymphatic hydrops
ES: Endolymphatic sac
FOV: Field of view
ICC: Intra-group correlation coefficient
MD: Ménière’s disease
MRI: Magnetic resonance imaging
PACS: Picture archiving and communication system
SCC: Semicircular canal
SR-PCI: Synchrotron phase contrast imaging
TE: Echo time
TR: Repetition time
VA: Vestibular aqueduct
